# A Mitogenomic Perspective on the Phylogenetic Position of the *Hapalogenys* Genus (Acanthopterygii: Perciformes) and the Evolutionary Origin of Perciformes

**DOI:** 10.1371/journal.pone.0103011

**Published:** 2014-07-31

**Authors:** Tao Wei, Yuena Sun, Bo Zhang, Rixin Wang, Tianjun Xu

**Affiliations:** Laboratory of Fish Biogenetics & Immune Evolution, College of Marine Science, Zhejiang Ocean University, Zhoushan, China; Institute of Biochemistry and Biology, Germany

## Abstract

The *Hapalogenys* genus was the most controversial and problematic in phylogenetic position of Percoidei. In this study, we rechecked the taxonomic status of *Hapalogenys* in Percoidei using complete mitochondrial genome data. We purposefully added a new complete mitochondrial sequence from chosen species of *Hapalogenys* and conducted phylogenetic analyses using a large complete mitochondrial data set. The resultant tree topologies were congruent from partitioned Bayesian and Maximum-likelihood methods. The results indicated that *Hapalogenys* was distantly related to Haemulidae and could be removed from Haemulidae. The results supported the *Hapalogeny* was upgraded to a family rank titled Hapalogenyidae, and it should be recognized in a separate family of Hapalogenyidae. A relaxed molecular-clock Bayesian analysis indicated that the divergence times of Perciformes groups had a much older than the available old fossil records. The origin of the common ancestral lineage of Perciformes fish was estimated in the late Jurassic about 149 Myr ago.

## Introduction

The largest order of vertebrate, the Perciformes is the most diversified teleost, and it has approximate 9000 extant species placed into 18 suborders. The most species of Perciformes occur in both marine and freshwater areas ranging from shallow freshwater to depths of more than 2300 metres in the ocean [1]. Owning to some species possessing scientific and economic importance to fishery, the Perciformes fish has gained much attention in the aspect of molecular phylogenetics and systematics [2], [3]. Currently, the Perciformes order is divided into 148 families, including about 1367 genera [1]. Among all the genera of the order, the *Hapalogenys* genus is one of the most controversial and problematic about its phylogenetic position in Percoidei. The genus contains about 12 recognized species, such as *Hapalogenys analis*, *Hapalogenys kishinouyei* and *Hapalogenys nigripinnis.* It has been traditionally classified in the Haemulidae family because of the presence of chin pores in the order Perciformes [4]. All of the species typical inhabit sandy bottoms or muddy rocky shores along the tropical and temperate coast regions of West Pacific. However, some species have become very difficult to be found so far.

**Figure 1:**
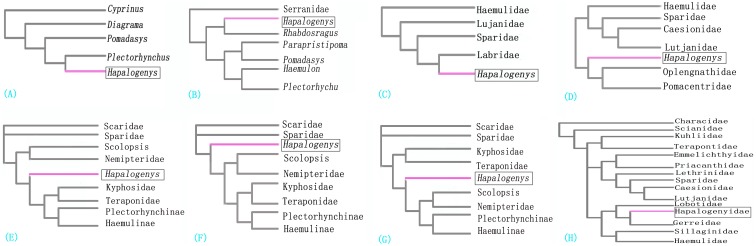
Alternative phylogenetic hypotheses between *Hapalogenys* and Percoidei family. (**A**) Parsimony and Neighbor-Joining tree for partial mtDNA cytb gene (Zhu et al., 2006), (**B**) Minimum-Evolution tree for mtDNA 16s RNA gene (Ren et al., 2007), (**C**) Neighbor-Joining tree for mtDNA 16s RNA gene (Ren and Zhang, 2007), (**D**) Neighbor-Joining tree for mtDNA cytb gene (Xu et al., 2010), (**E**) Bayesian, Maximux-likehood, and Parsimony tree for mtDNA 16s RNA gene (Liang et al., 2012), (**F**) Bayesian, Maximux-likehood, and Parsimony tree for nuclear TMO-4c4 gene (Liang et al., 2012), (**G**) Bayesian, Maximux-likehood, and Parsimony tree for mtDNA 16s RNA and nuclear TMO-4c4 gene (Liang et al., 2012), (**H**) Bayesian and Maximux-likehood tree for mtDNA 16s, COI, and Cytb gene (Tavera et al., 2012).

There has been some dispute in the literature as to genus *Hapalogenys* and whether it should be treated as separate families [4]–[6]. At the morphological level, the research of *Hapalogenys* is poorly understood, and its accurate taxomomic status remains ambiguity. Meanwhile, some evaluations of the classification and phylogeny of *Hapalogenys* have investigated the evolution of the complex genus. Different molecular markers, such as nuclear TMO-4c4 gene or mitochondrial genes (12s, 16s rRNA, COI and Cytb) have been used for evaluating phylogenetic relationships within the *Hapalogenys.* Some molecular examples of solving relationships of *Hapalogenys* include studies by Zhu *et al*. (2006), Ren *et al*. (2007), Ren and Zhang (2007), Liang *et al*. (2010), Xu *et al*. (2010), and Tavera *et al*. (2012) (Fig. 1) [6]–[11]. Each of these studies has provided valuable information for phylogenetic relationships of *Hapalogenys* fish. Zhu *et al.* (2006) proposed *Hapalogenys* belong to Haemulidae family, and it had a close genetic relationship with *Plectorhynchus*. However, later, Ren *et al* (2007) and Ren and Zhang’s (2007) results suggested the genus *Hapalogenys* was removed from the family Haemulidae according to their molecular findings. In 2010, Xu *et al.* advised that *Hapalogenys* had surpassed the genetic differentiation level with other Haemulidae species. However, it was still belong to Percoidea for the genetic distance and phylogenetic analysis. Liang *et al.* (2012) and Tavera *et al*. (2012) suggested *Hapalogenys* could potentially be removed from the Haemulidae family. The proposed hypotheses based on the short sequences were contradictory and controversial due to different analytical methods and datasets. The phylogeny of the *Hapalogenys* has never been studied by using a longer mitogenomic dataset and a robust phylogenetic tree with appropriate representation species. Recently, it has been demonstrated that the complete mitochondrial genomes can offer sufficient resolution for constructing a robust phylogeney and estimating divergence time events compared with single gene sequences or segments [12], [13]. Therefore, to make a further toward the comprehension for *Hapalogenys* phylogeny, it was appropriate to address these questions from a longer mitogenomic perspective.

The evolutionary history of the Perciformes fishes has received little attention oweing to scarce representation in the fossil record. Although some percoid family’s fossil was record [14], the evolutionary history and systematics of the Perciformes remained obscured. The recent technical developments in the molecular estimation of divergence times have provided calculable time scales for molecular phylogenetic trees. It can present a new approach to elaborate evolutionary history which can not be estimated by the fossil record alone [15], [16]. Therefore, we make the effort to generate new viewpoints to get insight into the origin time of Perciformes from time information of molecular data.

In this study¸ in order to address questions about the phylogenetic position of the *Hapalogenys* genus and the evolutionary origin of Perciformes, we obtained the complete mitochondrial genome sequence of a species of *Hapalogenys* for the first time. The unambiguously aligned mitochondrial genomes were used for implementing phylogenetic analysis to illustrate the phylogenetic position of the *Hapalogenys* genus in Percoidei. In addition, we estimated the divergence time of Perciformes using a relaxed molecular-clock method to clarify the time scale of evolution of the group.

## Materials and Methods

### Sampling of specimens and mitogenome sequencing

*Hapalogenys analis* was purchased at a local fish market (Zhoushan, China). Taxonomic status of the fishes was identified by morphology. The muscle tissue was excised and stored in 95% ethanol and frozen at −20°C. We determined the complete mitogenome sequences of *H. analis* and then combined with all previously published mitogenome sequences of Percoidei taxa (http://mitofish.aori.u-tokyo.ac.jp/).

### Molecular Methods

Total genomic DNA was extracted from the approximated 20 mg of tissues using the Tiangen DNA extraction kit according to the manufacturer’s protocol. PCR amplification and sequencing were performed as described in Wei *et al*. [17]. The sequencing reactions for each of the PCR product were implemented in both directions, and the sequences of overlapping fragments were checked and assembled to determine the complete mitochondrial genome sequence. The complete mitochondrial genome was sequenced using primers in Table S1. In addition, the taxa lists in this study were shown in Table S2 coupled with GenBank accession numbers.

### Sequence editing and alignment

The mitochondrial genome sequences obtained were edited and analyzed by using DNAstar and DNASIS 3.2 (Hitachi Software Engineering). We combined the newly determined sequences with 61 previously published mitogenomes to construct data sets. All sequences from the L-strand-encoded genes were switched to complementary strand sequences. Amino acid sequences were used for aligning protein-coding genes. After alignment, they were translated back to nucleotide sequences [18]. All positions comprised of gaps and stop codons were not including from the subsequent phylogenetic analysis. The ND6 gene was not applied to the phylogenetic analysis owing to its poor phylogenetic performance and heterogeneous base composition [19], [20]. The alignment of tRNA genes were performed according to the secondary structural model [18]. The 12S and 16S rRNA genes were firstly aligned using Mega 5.0 with default parameters [21] and then inspected manually using MacClade ver 4.08 for any misalignments [22]. In addition, the control region was excluded because positional homology was not determined in distantly related species. The final data set was comprised of 10476 positions from the first, second and third codon positions of the 12 protein-coding genes, 2349 positions from the two rRNA genes, and 1410 positions from the tRNA genes for phylogenetic analysis.

### Phylogenetic analysis

The phylogenetic trees were performed by using partitioned Maximum likelihood and Bayesian inference. The most suitable model of DNA substitution was chosen with the jModeltest program by the Akaike Information Criterion (AIC) [23]. The partitioned Bayesian phylogenetic analyses were conducted by using MrBayes 3.2 [24]. The Markov chain Monte Carlo (MCMC) analyses (with random starting trees) was run for 3 million generations with one cold and three heated chains. We performed two independent runs for 3 million generations, with tree sampling every 100 generations. Runs were stopped after the standard deviation of split frequencies fell below 0.01. In addition, we employed the above datasets to the partitioned Maximum-likelihood (ML) analysis using RAxMLHPC [25]. For the dataset, exploring of the best scoring ML tree and a rapid bootstrap analysis were investigated with a general time reversible nucleotide model (GTR) with sites following a discrete gamma distribution (Γ) and some sities invariable (I). Nodal support of the tree was performed by 1000 replications.

### Estimation of divergence times

A relaxed molecular clock Bayesian method used in MCMCTREE program of PAML was implemented for dating analysis [26]. All time constrains were used with a unit of 100 Ma (1 = 100 Ma), because some model components were scale-variant in the Bayesian analysis. Including multiple fossil-based calibration points (Table S3) were employed for diverse teleostean lineages [13], [15], [27], [28]. The independent-rates (IR) model was used for assigning the prior of rates within internal nodes. It has been thought that it is more appropriate than the autocorrelated-rates model in divergence time estimation [29]. In order to judge possible failure of the Markov chains to attain stationarity, at least two Markov chain Monte Carlo (MCMC) analyses were utilized with two different seeds for each analysis. Each MCMC analysis approximately with a burn-in period of 10,000 cycles was performed, and every 100 cycles was used for generating a total of 10,000 samples. The similar results were obtained and observed from the two runs.

## Results and Discussion

### Phylogenetic position of *Hapalogenys*

**Figure 2:**
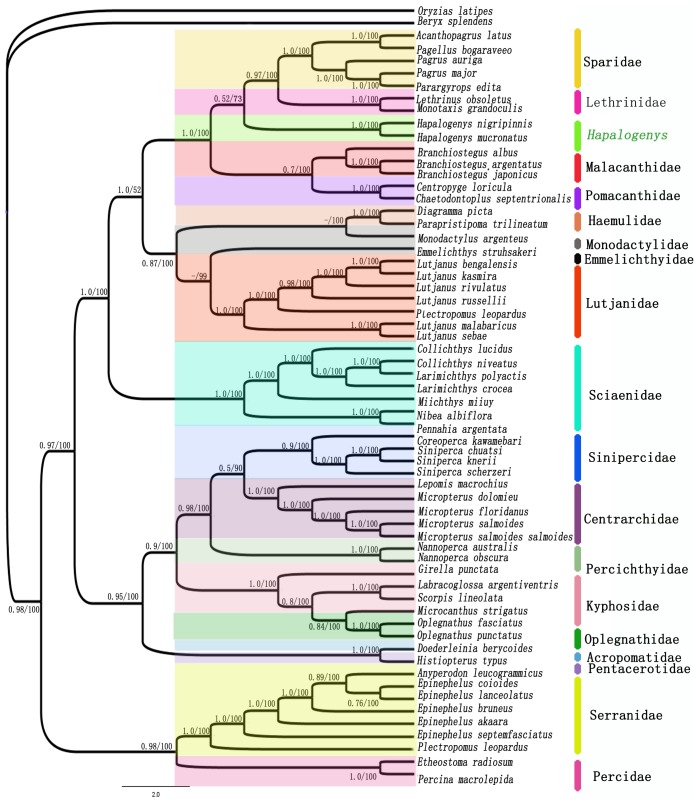
The majority rule consensus tree of *Hapalogenys* and related taxa are obtained from partitioned BI, ML analysis of mitochondrial genome dataset. Numbers near internal branches indicate the ML bootstrap support values (left) and Bayesian posterior probabilities (right), respectively. Support values less than 50% for the node are indicated by a dash.

The present study was based on the complete mitochondrial genome sequences from some chosen species which represented the diversity of the Perciformes species. Partitioned Maximum-likelihood and Bayesian analysis based on complete mitochondrial genome yielded essentially the same tree topology (Fig. 2). The resultant trees indicated that *Hapalogenys* was found to be monophyletic and had been most closely relationship with Lethrinidae. The *Hapalogenys* displayed a high degree of molecular divergence, which was similar to detected morphological difference. It was different from groups of the Haemulidae herein. It had been removed from Haemulidae family, and formed a distinct group outside the Haemulidae group. The Haemulidae was resolved as a sister group relative to Mondactylidae. It had a distant phylogenetic relationship with *Hapalogenys* genus.

The results of these analyses were different from the results of previous phylogenetic studies. Traditionally, the genus *Hapalogenys* was placed in the Haemulidae family among Perciformes owning to the presence of pores [30]. But Johnson (1984) took the genus as “incertae sedis” in the Percoidei due to its uncertain affinities [31]. Springer and Raasch proposed a new family name for the *Hapalogenys* in 1995, but there was not any strong supporting evidence for the genus [5]. McKay (2001) retained *Hapalogenys* genus in Haemulidae for convenience. In fact, he had also realized the genus should be removed from the Haemulidae [32]. However, the phylogenetic relationship of Pomadasyidae fish was analyzed using the cytochrome b genes molecular marker by Zhu *et al*. (2006) [6]. He proposed that *Hapalogenys* and *Plectorhynchus* had a close genetic relationship with *Pomadasys*. Significantly, the 16S rRNA gene datasets were used to infer phylogenetic relationships among different genera of Haemulidae, the results suggested the genus *Hapalogenys* was removed from the family Haemulidae and should be redefined [7], [8]. The most recent phylogenetic analysis of *Hapalogenys* also provided similar result by using partial mitochondrial and nuclear genes [10], [11]. However, deep nodes were weak in constructing phylogenetic trees. It indicated that the short sequence had difficult in resolving the particular phylogenetic problem.

The most recent morphological research of the phylogenetic position of *Hapalogenys* was derived from having the combination of characteristics [9]. The results indicated that *Hapalogenys* genus differed from the Haemulidae group at morphological level. But previous morphological studies had put forward the contrary, that *Hapalogenys* was more likely affiliated with Haemulidae family among Perciforms [1], [33]. The phylogenetic analyses of plentiful mitochondrial genome sequence data presented herein, and it suggested the genus *Hapalogenys* should be removed from Haemulidae. Our molecular data was inconsistent with traditional morphological data, but was agreed with some previous molecular hypotheses. According to these results, we supported that the *Hapalogenys* genus was upgraded to a family rank titled Hapalogenyidae and should be recognized in a separate family of Hapalogenyidae.

### Divergence Time Estimation

**Figure 3:**
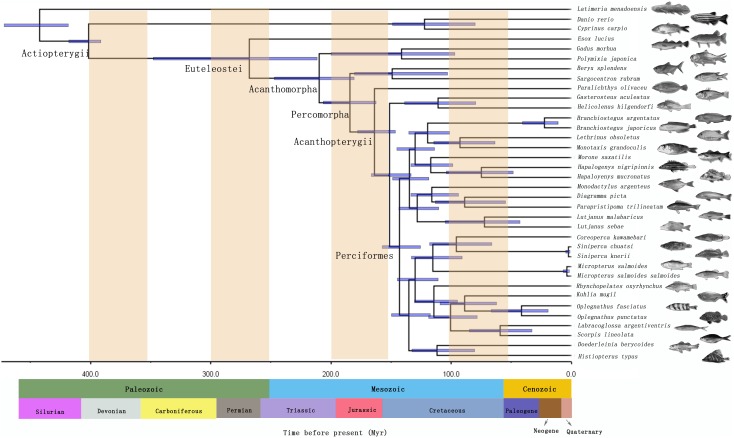
Posterior distributions of divergence times of Perciformes fishes and related species. Divergence times were estimated from the partitioned Bayesian analysis using PAML program package. The horizontal bars represent the estimated 95% credibility intervals of the divergence time estimation.

In present study, overall MCMCTREE analyses of the divergence time were based on the assumption of independent rates (IR). The relaxed molecular-clock Bayesian analysis of divergence time estimates suggested that the Perciformes fish was estimated to be diverged from the ancestral lineage of the Gasterosteiformes and Scorpaeniformes during the about 149 Ma with a credible interval of 133–166 Ma (Fig. 3). The common ancestor of the order has diverged into the different family on 145 Ma. Previous oldest Perciformes fossil was discovered from the early Eocene. Paleontological records indicated that Perciformes fish originated in the late Cretaceous about 90 Mya. Some families among Perciformes fastly radiated in the Paleocene or early Eocene after the Cretaceous/Tertiary boundary [34]. Benton (1993) analyzed the radiation of Perciformes fishes based on the oldest fossil records. The results showed common ancestor of the Perciformes fish dated back to over 149 Mya in the Jurassic. Meanwhile, according to Benton’s (1998) fossil records, it suggested that the radiation of Perciformes species traced back to the early Cenozoic [35], but the molecular clock estimation time take it out from the Jurassic to the early Cretaceous by Kumazawa *et al*. (1999) [36]. Cantatore *et al.* (1994) estimated divergence times of Perciformes fish based on mitochondrial Cytb genes by using molecular-clock approach. The acquired divergence times were similar to those in this study [37]. There seemed to be a significant time gap between divergence estimates from molecular evidence and the first occurrence evidence of fossil records. One explanation might be provided for apparent discrepancies between fossil chronologies and molecular data. In Perciformes families, some fossil records of fish were completely absence, and a number of the first occurrences with recognizable fossil records were based merely on the otoliths [36], [38]. Meanwhile, owning to the less preservable nature of fish fossils, disappearance or occurrence of fish was inclined to be affected by limited fossil localities of unusual preservation [35]. These questions might lead to underestimate origination of fish, and cause prejudice in deducing evolutionary history of Perciformes fishes. So far, some studies about the molecular divergence time estimates exceeded fossil record have also been found in vertebrates [16], [39], [40]. Therefore, we considered that the lacking of teleostean fossil record might lead to the discrepancy between fossil and molecular dates, rather than the inaccuracy of our molecular time estimates. Of particular interest for divergence time studies, the common ancestral lineage of Perciformes fish originated in the late Jurassic about 149 Ma (Fig. 3), and it would undergo the Cretaceous mass extinction events. Major climatic and ecosystems changes occurred during Cretaceous time resulted in extinction of most archaic actinopterygian groups. The survival of the ancestor of Perciformes through the extinction events resulted in the subsequent familial radiation during the Cretaceous-Tertiary. These time estimates were roughly in accordance with those of the previous molecular study [36]. Our results proposed that a large wave of familial radiation for Perciformes occurred during the Cretaceous-Tertiary. More studies are required to resolve the relationships between Perciformes fish radiations and global changes at the Cretaceous-Tertiary.

## Supporting Information

Table S1 **Primer locations and sequences.**
(DOC)

Table S2 **List of the species used in this study with DDBJ/EMBL/GenBank accession numbers.**
(DOC)

Table S3 **List of time constraints used in divergence time estimation.**
(DOC)
