# Supplementary material for: A Mitogenomic Perspective on the Phylogenetic Position of the Hapalogenys Genus (Acanthopterygii: Perciformes) and the Evolutionary Origin of Perciformes
Source: PLoS One. 2014 Jul 31;9(7):e103011. doi: 10.1371/journal.pone.0103011 (PMC4117523; doi:10.1371/journal.pone.0103011)
Supplement: Table S1 — Primer locations and sequences. (DOC) [file pone.0103011.s001.doc]

**Table S1.** Primer locations and sequences

| No. | Upstream primer | Upstream primer sequences | Downstream primer | Downstream primer sequence |
| --- | --- | --- | --- | --- |
| 1 | L31 | GAAGATGTTAGGATAGGCCCTGGAAAG | H5577 | GGGTTGAATAAGTTTGGGTTGGATGAG |
| 2 | L4917 | TACGCCATAGCCCTCACA | H5804 | CCTGACGAGGCAAGTAGA |
| 3 | L5887 | CAGGGGCATCTGTAAACT | H8035 | GCTCATTAGGAAAAGAGTG |
| 4 | L5982 | ATAAAACCACCACCAATTTCACAGTAC | H15653 | GAGTGAGTCGAGGGGTTTCAAAGAGTA |
| 5 | L8117 | TCTACCGTGGGTCTTGTT | H8989 | GGAGTGCGATGGCCTTGA |
| 6 | L9794 | TTCCGTTCTCTATGCGATT | H10600 | GCTCTTGTTTGGTGTGGTT |
| 7 | L10117 | GCTCGCATTCCACCGAACCCATCTACTGT | H15608 | CTAGCTTTGGGAGTCAGGGGTGAATCGTC |
| 8 | L10763 | GAGGGAACCAGGCGGAACGGCTAAACG | H16395 | GGGCACAACCATAGGAATGTGAGGACA |
| 9 | L14333 | GTGACTTGAAAAACCACCGTT | H15550 | CTCCATCTCCGGTTTACAA |
| 10 | L15540 | AGCACCGGTCTTGTAAACCG | H30 | GGGCTCATCTTAACATCTTCA |
