# Supplementary material for: A Mitogenomic Perspective on the Phylogenetic Position of the Hapalogenys Genus (Acanthopterygii: Perciformes) and the Evolutionary Origin of Perciformes
Source: PLoS One. 2014 Jul 31;9(7):e103011. doi: 10.1371/journal.pone.0103011 (PMC4117523; doi:10.1371/journal.pone.0103011)
Supplement: Table S2 — List of the species used in this study with DDBJ/EMBL/GenBank accession numbers. (DOC) [file pone.0103011.s002.doc]

**Table S2**. List of the species used in this study with DDBJ/EMBL/GenBank accession numbers.

| Order | Family | Species | Accession Number |
| --- | --- | --- | --- |
| **Carcharhiniformes** | Scyliorhinidae | *Scyliorhinus canicula* | [Y16067](http://www.ncbi.nlm.nih.gov/nuccore/Y16067) |
| **Beryciformes** | Holocentridae | *Sargocentron rubrum* | [AP004432](http://www.ncbi.nlm.nih.gov/nuccore/AP004432) |
|  | Berycidae | *Beryx splendens* | [AP002939](http://www.ncbi.nlm.nih.gov/nuccore/AP002939) |
| **Gasterosteiformes** | Gasterosteidae | *Gasterosteus aculeatus* | [AP002944](http://www.ncbi.nlm.nih.gov/nuccore/AP002944) |
| **Scorpaeniformes** | Sebastidae | *Helicolenus hilgendorfi* | [AP002948](http://www.ncbi.nlm.nih.gov/nuccore/AP002948) |
| **Cypriniformes** | Cyprinidae | *Danio rerio* | [AC024175](http://www.ncbi.nlm.nih.gov/nuccore/AC024175) |
|  |  | *Cyprinus carpio* | X61010 |
| **Pleuronectiformes** | Paralichthyidae | *Paralichthys olivaceus* | [AB028664](http://www.ncbi.nlm.nih.gov/nuccore/AB028664) |
| **Beloniformes** | Adrianichthyidae | *Oryzias latipes* | [AP004421](http://www.ncbi.nlm.nih.gov/nuccore/AP004421) |
| **Esociformes** | Esocidae | *Esox lucius* | [AP004103](http://www.ncbi.nlm.nih.gov/nuccore/AP004103) |
| **Gadiformes** | Gadidae | *Gadus morhua* | [X99772](http://www.ncbi.nlm.nih.gov/nuccore/X99772) |
| **Polymixiiformes** | Polymixiidae | *Polymixia japonica* | AB034826 |
| **Coelacanthiformes** | Coelacanthidae | *Latimeria menadoensis* | [AP006858](http://www.ncbi.nlm.nih.gov/nuccore/AP006858) |
| **Perciformes** | Malacanthidae | *Branchiostegus albus* | [EU861053](http://www.ncbi.nlm.nih.gov/nuccore/EU861053) |
|  |  | *Branchiostegus argentatus* | [EU861054](http://www.ncbi.nlm.nih.gov/nuccore/EU861054) |
|  |  | *Branchiostegus japonicus* | [EU861052](http://www.ncbi.nlm.nih.gov/nuccore/EU861052) |
|  | Pomacanthidae | *Centropyge loricula* | [AP006006](http://www.ncbi.nlm.nih.gov/nuccore/AP006006) |
|  |  | *Chaetodontoplus septentrionalis* | [AP006007](http://www.ncbi.nlm.nih.gov/nuccore/AP006007) |
|  | Sciaenidae | *Collichthys lucidus* | [HM447239](http://www.ncbi.nlm.nih.gov/nuccore/HM447239) |
|  |  | *Collichthys niveatus* | [HM219223](http://www.ncbi.nlm.nih.gov/nuccore/HM219223) |
|  |  | *Larimichthys crocea* | [EU339149](http://www.ncbi.nlm.nih.gov/nuccore/EU339149) |
|  |  | *Larimichthys polyactis* | [FJ618559](http://www.ncbi.nlm.nih.gov/nuccore/FJ618559) |
|  |  | *Pennahia argentata* | [HQ890946](http://www.ncbi.nlm.nih.gov/nuccore/HQ890946) |
|  |  | *Nibea albiflora* | [HQ890947](http://www.ncbi.nlm.nih.gov/nuccore/HQ890947) |
|  |  | *Miichthys miiuy* | [HM447240](http://www.ncbi.nlm.nih.gov/nuccore/HM447240) |
|  | Haemulidae | *Diagramma picta* | [AP009167](http://www.ncbi.nlm.nih.gov/nuccore/AP009167) |
|  |  | *Hapalogenys* *analis* | NC_019646* |
|  |  | *Hapalogenys nigripinnis* | [HM754620](http://www.ncbi.nlm.nih.gov/nuccore/HM754620) |
|  |  | *Parapristipoma trilineatum* | [AP009168](http://www.ncbi.nlm.nih.gov/nuccore/AP009168) |
|  | Acropomatidae | *Doederleinia berycoides* | [AP009181](http://www.ncbi.nlm.nih.gov/nuccore/AP009181) |
|  | Emmelichthyidae | *Emmelichthys struhsakeri* | [AP004446](http://www.ncbi.nlm.nih.gov/nuccore/AP004446) |
|  | Serranidae | *Epinephelus akaara* | [EU043377](http://www.ncbi.nlm.nih.gov/nuccore/EU043377) |
|  |  | *Epinephelus bruneus* | [FJ594964](http://www.ncbi.nlm.nih.gov/nuccore/FJ594964) |
|  |  | *Epinephelus coioides* | [EU043376](http://www.ncbi.nlm.nih.gov/nuccore/EU043376) |
|  |  | *Epinephelus lanceolatus* | [FJ472837](http://www.ncbi.nlm.nih.gov/nuccore/FJ472837) |
|  |  | *Epinephelus septemfasciatus* | FJ594966 |
|  |  | *Plectropomus leopardus* | [DQ101270](http://www.ncbi.nlm.nih.gov/nuccore/DQ101270) |
|  |  | *Anyperodon leucogrammicus* | [GQ131336](http://www.ncbi.nlm.nih.gov/nuccore/GQ131336) |
|  | Percidae | *Etheostoma radiosum* | [AY341348](http://www.ncbi.nlm.nih.gov/nuccore/AY341348) |
|  |  | *Percina macrolepida* | [DQ536430](http://www.ncbi.nlm.nih.gov/nuccore/DQ536430) |
|  | Pentacerotidae | *Histiopterus typus* | [AP006807](http://www.ncbi.nlm.nih.gov/nuccore/AP006807) |
|  | Kyphosidae | *Kyphosus cinerascens* | [AP011061](http://www.ncbi.nlm.nih.gov/nuccore/AP011061) |
|  |  | *Labracoglossa argentiventris* | [AP011062](http://www.ncbi.nlm.nih.gov/nuccore/AP011062) |
|  |  | *Microcanthus strigatus* | [AP006009](http://www.ncbi.nlm.nih.gov/nuccore/AP006009) |
|  |  | *Girella punctata* | [AP011060](http://www.ncbi.nlm.nih.gov/nuccore/AP011060) |
|  |  | *Scorpis lineolata* | [AP011064](http://www.ncbi.nlm.nih.gov/nuccore/AP011064) |
|  | Lethrinidae | *Lethrinus obsoletus* | [AP009165](http://www.ncbi.nlm.nih.gov/nuccore/AP009165) |
|  |  | *Monotaxis grandoculis* | [AP009166](http://www.ncbi.nlm.nih.gov/nuccore/AP009166) |
|  | Lutjanidae | *Lutjanus bengalensis* | [FJ171339](http://www.ncbi.nlm.nih.gov/nuccore/FJ171339) |
|  |  | *Lutjanus kasmira* | [FJ416614](http://www.ncbi.nlm.nih.gov/nuccore/FJ416614) |
|  |  | *Lutjanus malabaricus* | [FJ824741](http://www.ncbi.nlm.nih.gov/nuccore/FJ824741) |
|  |  | *Lutjanus rivulatus* | [AP006000](http://www.ncbi.nlm.nih.gov/nuccore/AP006000) |
|  |  | *Lutjanus russellii* | [EF514208](http://www.ncbi.nlm.nih.gov/nuccore/EF514208) |
|  |  | *Lutjanus sebae* | [FJ824742](http://www.ncbi.nlm.nih.gov/nuccore/FJ824742) |
|  |  | *Pletropomus leopardus* | DQ101270 |
|  | Centrarchidae | *Micropterus dolomieu* | [AB378749](http://www.ncbi.nlm.nih.gov/nuccore/AB378749) |
|  |  | *Micropterus floridanus* | [HQ391897](http://www.ncbi.nlm.nih.gov/nuccore/HQ391897) |
|  |  | *Micropterus salmoides* | [DQ536425](http://www.ncbi.nlm.nih.gov/nuccore/DQ536425) |
|  |  | *Micropterus salmoides salmoides* | [HQ391896](http://www.ncbi.nlm.nih.gov/nuccore/HQ391896) |
|  |  | *Lepomis macrochirus* | [JN389795](http://www.ncbi.nlm.nih.gov/nuccore/JN389795) |
|  | Monodactylidae | *Monodactylus argenteus* | [AP009169](http://www.ncbi.nlm.nih.gov/nuccore/AP009169) |
|  | Percichthyidae | *Nannoperca australis* | [JF519732](http://www.ncbi.nlm.nih.gov/nuccore/JF519732) |
|  |  | *Nannoperca obscura* | [JF519733](http://www.ncbi.nlm.nih.gov/nuccore/JF519733) |
|  | Oplegnathidae | *Oplegnathus fasciatus* | [DQ872160](http://www.ncbi.nlm.nih.gov/nuccore/DQ872160) |
|  |  | *Oplegnathus punctatus* | [AP011066](http://www.ncbi.nlm.nih.gov/nuccore/AP011066) |
|  | Sparidae | *Pagellus bogaraveo* | [AB305023](http://www.ncbi.nlm.nih.gov/nuccore/AB305023) |
|  |  | *Pagrus auriga* | [AB124801](http://www.ncbi.nlm.nih.gov/nuccore/AB124801) |
|  |  | *Pagrus major* | [AP002949](http://www.ncbi.nlm.nih.gov/nuccore/AP002949) |
|  |  | *Parargyrops edita* | [EF107158](http://www.ncbi.nlm.nih.gov/nuccore/EF107158) |
|  |  | *Acanthopagrus latus* | [EF506764](http://www.ncbi.nlm.nih.gov/nuccore/EF506764) |
|  | Sinipercidae | *Siniperca chuatsi* | [JF972568](http://www.ncbi.nlm.nih.gov/nuccore/JF972568) |
|  |  | *Siniperca knerii* | [JN378751](http://www.ncbi.nlm.nih.gov/nuccore/JN378751) |
|  |  | *Siniperca scherzeri* | [JN084101](http://www.ncbi.nlm.nih.gov/nuccore/JN084101) |
|  |  | *Coreoperca kawamebari* | [AP005990](http://www.ncbi.nlm.nih.gov/nuccore/AP005990) |

* New sequence in this study
