# Supplementary material for: A Mitogenomic Perspective on the Phylogenetic Position of the Hapalogenys Genus (Acanthopterygii: Perciformes) and the Evolutionary Origin of Perciformes
Source: PLoS One. 2014 Jul 31;9(7):e103011. doi: 10.1371/journal.pone.0103011 (PMC4117523; doi:10.1371/journal.pone.0103011)
Supplement: Table S3 — List of time constraints used in divergence time estimation. (DOC) [file pone.0103011.s003.doc]

**Table S3.** List of time constraints used in divergence time estimation

| **Node** | **Constraints** | **Calibration information** |
| --- | --- | --- |
| **A** | **U 472** | The minimum mage for the basal split of bony fish based on the earliest known acanthodian remains form Late Ordovician (Azuma et al., 2008) |
|  | **L 419** | The Psarolepis fossil from Ludlow (Silurian) (Azuma et al., 2008) |
| **B** | **U419** | The minimum age for the Sarcopterygii/ Actinopterygii split (Miya et al., 2010). |
|  | **L392** | The Moythomasia fossil (actinopteran) from the Givetian/Eifelian boundary (Miya et al., 2010). |
| **C** | **U148** | Estimated divergence time between Cyprinidae and [Balitoridae](http://fishbase.sinica.edu.tw/Summary/FamilySummary.php?ID=126) (Miya et al., 2010) |
|  | **L49** | The Parabarbus fossil (Cyprinidae) from the Ypresian (Tertiary) (Nakatani et al., 2011) |
| **D** | **L74** | The Esteseox foxi fossil (Esociformes) from the Campanian (Cretaceous) (Miya et al., 2010) |
| **E** | **L94** | The Berycopsis fossil (Polymixiidae) from the Cenomanian (Cretaceous)^a^ |
| **F** | **L50** | The pleuronectiform fossil from the Ypresian (Tertiary)^a^ |
| **G** | **L40** | The Perciformes fossil from Lutetian (Eocene)^a^ |

a: Minima (L) are based on earliest occurrences in the fossil records (Benton, 1993)
